# Contribution of molecular analysis to the typification of the non-functioning pituitary adenomas

**DOI:** 10.1371/journal.pone.0180039

**Published:** 2017-07-10

**Authors:** Laura Sanchez-Tejada, Ruth Sanchez-Ortiga, Cristina Lamas, Rosa Camara, Pedro Riesgo, Carmen Fajardo, Francisco Ignacio Aranda, Antonio Pico

**Affiliations:** 1 Research Unit, Institute for Health and Biomedical Research (Isabial-Fisabio Foundation), Hospital General Universitario de Alicante, Alicante, Spain; 2 Endocrinology Department, Hospital General Universitario de Alicante, Alicante, Spain; 3 Endocrinology Department, Hospital General Universitario de Albacete, Albacete, Spain; 4 Endocrinology Department, Hospital Universitario y Politecnico La Fe, Valencia, Spain; 5 Neurosurgery Department, Hospital de La Ribera, Alzira, Valencia, Spain; 6 Endocrinology Department, Hospital de La Ribera, Alzira, Spain; 7 Pathology Department, Hospital General Universitario de Alicante, Alicante, Spain; University of Cordoba, SPAIN

## Abstract

**Aim:**

The WHO Classification of Tumours of Endocrine Organs considers the inmunohistochemical characterization of pituitary adenomas (PA) as mandatory for patient diagnosis. Recent advances in the knowledge of the molecular patterns of these tumours could complement this classification with gene expression profiling.

**Methods:**

Within the context of the Spanish Molecular Registry of Pituitary Adenomas (REMAH), a multicentre clinical-basic research project, we analysed the molecular phenotype of 142 PAs with complete IHC and clinical information. Gene expression levels of all pituitary hormones, type 1 corticotrophin-releasing hormone receptor, dopamine receptors and arginine vasopressin receptor 1b were measured by quantitative real-time polymerase chain reaction. In addition, we used three housekeeping genes for normalization and a pool of nine healthy pituitary glands from autopsies as calibration reference standard.

**Results:**

Based on the clinically functioning PA (FPA: somatotroph, corticotroph, thyrotroph and lactotroph adenomas), we established the interquartile range of relative expression for all genes studied in each PA subtype. That allowed molecularly the different PA subtypes, including the clinically non-functioning PA (NFPA). Afterwards, we estimated the concordance of the molecular and immunohistochemical classification with clinical diagnosis in FPA and between them in NFPA. The kappa values were higher in molecular than in immunohistochemical classification in FPA and showed a bad concordance in all NFPA subtypes.

**Conclusions:**

According to these results, the molecular characterization of the PA complements the IHC analysis, allowing a better typification of the NFPA.

## Introduction

Pituitary adenomas (PA) constitute 10%–15% of intracranial neoplasms. They are currently classified according to their clinical, biochemical, radiological and inmunohistochemical (IHC) characteristics as well as their tumour behaviour [1, 2, 3].

Clinically, PAs are divided into two main types: functioning pituitary adenomas (FPAs) and non-functioning pituitary adenomas (NFPAs). FPAs are accompanied by a clinical syndrome related to hormone hypersecretion: Cushing’s disease, acromegaly, galactorrhea-hypogonadism or hyperthyroidism. NFPAs include a cluster of pituitary tumours without endocrine manifestations of hormone overproduction, which are diagnosed incidentally or due to neuro-ophthalmological symptoms. They comprise approximately 30%–35% of pituitary tumours [4, 5].

Radiologically, two classification systems are currently used. The Hardy classification divides PAs into four grades based on their size and the invasiveness in the sella turcica [6]. The Knops classification takes into consideration the tumour invasion of the cavernous sinus according to coronal sections of magnetic resonance imaging (MRI) scans, with the readily detectable internal carotid artery serving as the radiological landmark [7].

Pathologically, different proposals have aimed to classify pituitary tumours over the years, ranging from the cellular staining properties to the latest World Health Organization (WHO) classification based on IHC and ultrastructural criteria [8]. This classification recognises five PA subtypes, depending on the aberrant hormone immunostaining: prolactin tumour (PRL), growth hormone tumour (GH), adrenocorticotropic hormone tumour (ACTH), thyroid-stimulating hormone tumour (TSH, α-subunit (SU)) and gonadotroph hormone tumour (follicle-stimulating hormone (FSH), luteinising hormone (LH) or SU) [8]. Among these, those non-gonadotroph and non-functioning tumours are known as silent (S) adenomas. Some of them are multihormonal. Finally, pituitary adenomas without IHC-detected aberrant hormone production are called null cell (NC) adenomas [3, 9].

Currently, translational research is based mainly on genetic and epigenetic characteristics. As experience accumulates in this field, molecular studies will gradually become integrated into the standardised clinical management of PA, similarly to other tumours [10,11].

Silent adenomas have been attributed to the release of pituitary hormone isoforms without biological activity or to the reduced or absent secretion of the normal pituitary hormones [3]. Null cell adenomas show ultrastructural features of a particular pituitary cell type or express cell specific transcription factors but do not secrete proteins. In these scenarios, the IHC technique is unable to define the cellular origin of the tumour. Conversely, the mRNA analysis of pituitary hormones or their specific receptors could identify the specific cell clone from which the tumour arises, allowing a molecular typification of these tumours. The clinical implications of this knowledge have yet to be clarified.

The aim of this study was to characterize molecularly the silent and immunohistochemically null cell adenomas in a large series of pituitary adenomas and to study the concordance between the IHC and molecular typification of PA.

## Matherial and methods

The study was performed within the context of the Spanish Molecular Registry of Pituitary Adenomas (REMAH), a Spanish Multicentre Clinical-Basic Project [12]. The study complies with the Declaration of Helsinki and other applicable laws and received approval from the Local Ethics Committee (CEIC Hospital General Universitario de Alicante). None of the donors was from a vulnerable population and all donors or next of kin provided written informed consent that was freely given. One of the patients was 14 years old boy and his parents signed his informed consent.

### Participants

We collected 142 PA specimens with complete clinical and IHC data and sufficient quantity and quality of RNA for molecular analysis. Additionally, we obtained nine normal pituitary glands from autopsies and used them as calibration reference samples.

The tumours came from four university referral hospitals for pituitary surgery. Clinical, pathological, and radiological data were collected anonymously for each sample from the REMAH database. The information retrieved included sex; age; endocrine syndrome (Cushing's syndrome, acromegaly, hyperthyroidism or amenorrhea-hypogonadism); hormonal data; previous medical, surgical or radiotherapy treatment; tumour maximum diameter (TMD); radiological MRI extension of the tumour, and IHC results. Tumours were classified as invasive (Hardy’s grade IV) or non-invasive (Hardy’s grades I–III). We present the clinical baseline characteristics in Table 1.

**Table 1:** Clinical Baseline characteristics of patients.

**Age** (years)		51 ± 15
**Women**		78/142 (54.9%)
**Largest diameter of tumour** (mm)		23.1±12.4
Second surgery*		19/142 (13.4%)
**Radiotherapy**		0
**Medical treatment**		
ST adenomas		26/36 (72.2%)
		18 SSa
		3 SSa + DA
		2 SSa + Peg
		2 DA
		1 Peg
		8 without treatment
		2 unknown
TT adenomas		2/2 (100%)
		1 SSa
		1 carbimazole
CT adenomas		11/21 (52.4%)
		6 KET
		4 KET + DA
		1 DA
		9 without treatment
		1 unknown
LT adenomas		8/9 (88.9%)
		8 DA
		1 unknown
NFPA		10/74 (13.5%)
		10 DA
		61 without treatment
		3 unknown
**Invasiveness**	**Extension** (Hardy)	
Non Invasive	Intrasellar (I-II)	39/137 (28.5%)
	Extrasellar (III)	44/137 (32.1%)
Invasive	Invasive (IV)	54/137 (39.4%)
Unknown extension		5/142

Data shown as mean ± SD or n/total (%).ST: somatotroph, TT: thyrotroph adenomas, CT: corticotroph, LT: lactotroph adenomas, NFPA: non-functioning pituitary adenomas, SSa: somatostatin analogs, DA: dopamine agonist, Peg: Pegvisomant, KET: Ketoconazole.

### Clinical identification of pituitary adenomas

All cases presenting clinical and biochemical characteristics of a recognised endocrine syndrome were classified as FPA. We considered participants to have NFPA when they did not present any clinical or biochemical characteristics of a recognised endocrine syndrome and were diagnosed according to neuro-ophthalmological or radiological signs.

### IHC identification of NFPA

According to the IHC data, we defined the following subtypes of NFPA: 1) IHC gonadotroph adenomas (IHC-GT): tumours with positive immunostaining for LH, FSH or SU; 2) IHC silent corticotroph adenomas (IHC-sCT): tumours with positive immunostaining for ACTH without Cushing’s syndrome; 3) IHC silent somatotroph adenomas (IHC-sST): tumours with positive immunostaining for GH without acromegaly; 4) IHC silent thyrotroph adenomas (IHC-sTT): tumours with positive immunostaining for TSH without hyperthyroidism; 5) IHC silent lactotroph adenomas (IHC-sLT): tumours with positive immunostaining for PRL without clinical amenorrhea-hypogonadism; 6) IHC silent multihormonal adenomas (IHC-sMHA): tumours with positive immunostaining for more than one hormone without predominance of one of them, not associated to a clinical syndrome; 7) IHC null cell adenomas (IHC-NC): tumours without positive immunostaining for any pituitary hormone.

### Molecular identification of pituitary adenomas

First, we defined the interquartile ranges (IQR) of gene expression for each clinically diagnosed FPA subtype (Tables 2 and 3). Afterward, we assessed all cases with clinical diagnosis of NFPA (74/142) individually and reclassified them according to their dominant gene expression, taking as a reference the 25^th^ percentile of their corresponding FPA, as follows: 1) molecular gonadotroph adenomas (M-GT): tumours with a dominant expression of LH, FSH or S; 2) molecular silent corticotroph adenomas (M-sCT): tumours with a dominant gene expression of POMC, AVPR1b and CRH-R1 without Cushing syndrome [13,14]; 3) molecular silent somatotroph adenomas (M-sST): tumours with a dominant gene expression of GH without acromegaly syndrome; 4) molecular silent thyrotroph adenomas (M-sTT): tumours with dominant expression of TSH without hyperthyroidism; 5) molecular silent lactotroph adenomas (M-sLT): dominant expression of PRL without amenorrhea-hypogonadism syndrome; 6) molecular silent multihormonal adenomas (M-sMHA): tumours with a dominant expression for more than one hormone without predominance of any of them, not associated to a clinical syndrome; 7) molecular null cell adenomas (M-NC): tumours that do not express any pituitary hormone gene or their receptors.

**Table 2:** Molecular classification of Pituitary Adenomas (PA).

Subtype PA		Dominant Gene Expression	Clinical symptoms
**CT adenomas**		*POMC*, *AVPR1 and CRH-R1*	Cushing’s syndrome
**ST adenomas**	Pure	*GH*	Acromegaly
	Mixed	*GH*, *PRL*	
	Plurihormonal		
**LT adenomas**		*PRL*	Galactorrhea or hypogonadism
**TT adenomas**		*TSH*	Hyperthyroidism
**GT adenomas**		*FSH*, *LH or α-subunit*	Non-Functioning PA
	FSHomas	*FSH*	
	LHomas	*LH*	
	Mixed	Combinations of *FSH*, *LH and α-Subunit*	
**Silent CT adenomas**		*POMC*, *AVPR1 and CRH-R1*	
**Silent ST adenomas**		*GH*	
**Silent LT adenomas**		*PRL*	
**Silent TT adenomas**		*TSH*	
**Null Cell Adenomas**		Without any hormone’s gene expression	
**Multihormonal**		Combinations of different hormones	

Pituitary subtypes (ST: somatotroph, TT: thyrotroph, CT: corticotroph, LT: lactotroph adenomas, GT: gonadotroph). Pituitary hormone genes (*FSH*: Follicle Stimulating Hormone; *LH*: Luteal Hormone; *CGA*: Gene encoding alpha subunit; *POMC*: Proopiomelanocortin (ACTH precursor); *GH*: Growth hormone; *PRL*: prolactin; *TSH*: thyroid stimulating hormone), and receptors involved in the synthesis and secretion of ACTH (*AVPR1b*: Vasopressin Receptor 1b; *CRH-R1*: Corticotropin Releasing Hormone Receptor 1).

**Table 3:** Ranges of relative expression (p25-p75) of the pituitary genes.

Subtypes	*FSH*	*LH*	*CGA*	*POMC*	*AVPR1*	*CRH-R1*	*GH*	*PRL*	*TSH*
**Null Cell**	0.00–0.16	0.00–0.07	0.00–0.03	0.00–0.03	0.01–0.55	0.06–0.88	0.01–0.05	0.00–0.02	0.00–0.01
**GT**	0.24–5.33	0.00–0.10	0.00–0.07	0.00–0.00	0.00–0.06	0.06–0.26	0.00–0.00	0.00–0.00	0.00–0.00
** FSHoma**	**0.75–6.87**	0.00–0.02	0.00–0.03	0.00–0.00	0.00–0.06	0.07–0.32	0.00–0.00	0.00–0.00	0.00–0.00
** LHoma**	0.07–0.34	**0.07–0.17**	0.00–0.00	0.00–0.03	0.03–0.08	0.06–0.17	0.00–0.00	0.00–0.00	0.00–0.00
** Mixed**	**0.05–0.65**	**0.02–0.21**	**0.05–0.31**	0.00–0.00	0.00–0.05	0.04–0.25	0.00–0.00	0.00–0.00	0.00–0.00
**CT Functioning**	0.00–0.21	0.00–0.31	0.00–0.24	**0.81–5.71**	**5.90–46.16**	**2.61–46.99**	0.00–0.17	0.00–0.50	0.00–0.00
** Silent**	0.00–0.07	0.00–0.01	0.00–0.17	**0.06–6.84**	**23.10–95.78**	**0.28–33.34**	0.00–0.04	0.00–0.02	0.00–0.01
**ST**	0.00–0.14	0.00–0.02	0.00–0.17	0.00–0.04	0.02–0.29	0.01–0.85	**0.65–2.97**	0.00–0.38	0.00–0.01
** Pure**	0.00–0.03	0.00–0.01	0.00–0.06	0.00–0.01	0.00–0.11	0.00–0.15	**0.58–3.51**	0.00–0.04	0.00–0.01
** Mixed**	0.00–0.01	0.00–0.00	0.11–0.26	0.00–0.01	0.01–0.04	0.02–0.13	**0.70–1.13**	**0.96–1.07**	0.00–0.00
**LT**	0.00–0.00	0.00–0.02	0.00–0.01	0.00–0.01	0.00–0.12	0.02–0.78	0.00–0.01	**0.01–2.00**	0.00–0.00
**TT Functioning**	0.00–0.00	0.00–0.00	**0.69–7.45**	0.00–0.00	0.00–0.00	0.00–0.00	0.02–0.04	0.00–0.00	**0.16–2.96**
** Silent**	0.01–0.22	0.00–0.00	**0.16–0.64**	0.00–0.03	0.00–0.16	0.05–0.50	0.00–0.01	0.00–0.00	**0.45–2.61**

PA subtype (GT: gonadotroph adenomas, CT: corticotroph adenomas, ST: somatotroph adenomas, LT: lactotroph adenomas, TT: thyrotroph adenomas). Pituitary hormone genes (*FSH*: Follicle Stimulating Hormone; *LH*: Luteal Hormone; *CGA*: Gene encoding alpha subunit; *GH*: Growth hormone; *POMC*: Proopiomelanocortin (ACTH precursor), *PRL*: prolactin, *TSH*: thyroid stimulating hormone), and receptors involved in the synthesis and secretion of ACTH (*AVPR1b*: Vasopressin Receptor 1b; *CRH-R1*: Corticotrophin Releasing Hormone Receptor 1). We cannot propose ranges of expression for silent lactotroph adenoma because we have only one possible tumour. In the same way, we cannot propose ranges of expression for silent somatotroph adenomas because there was none in our series.

**Fig 1:**
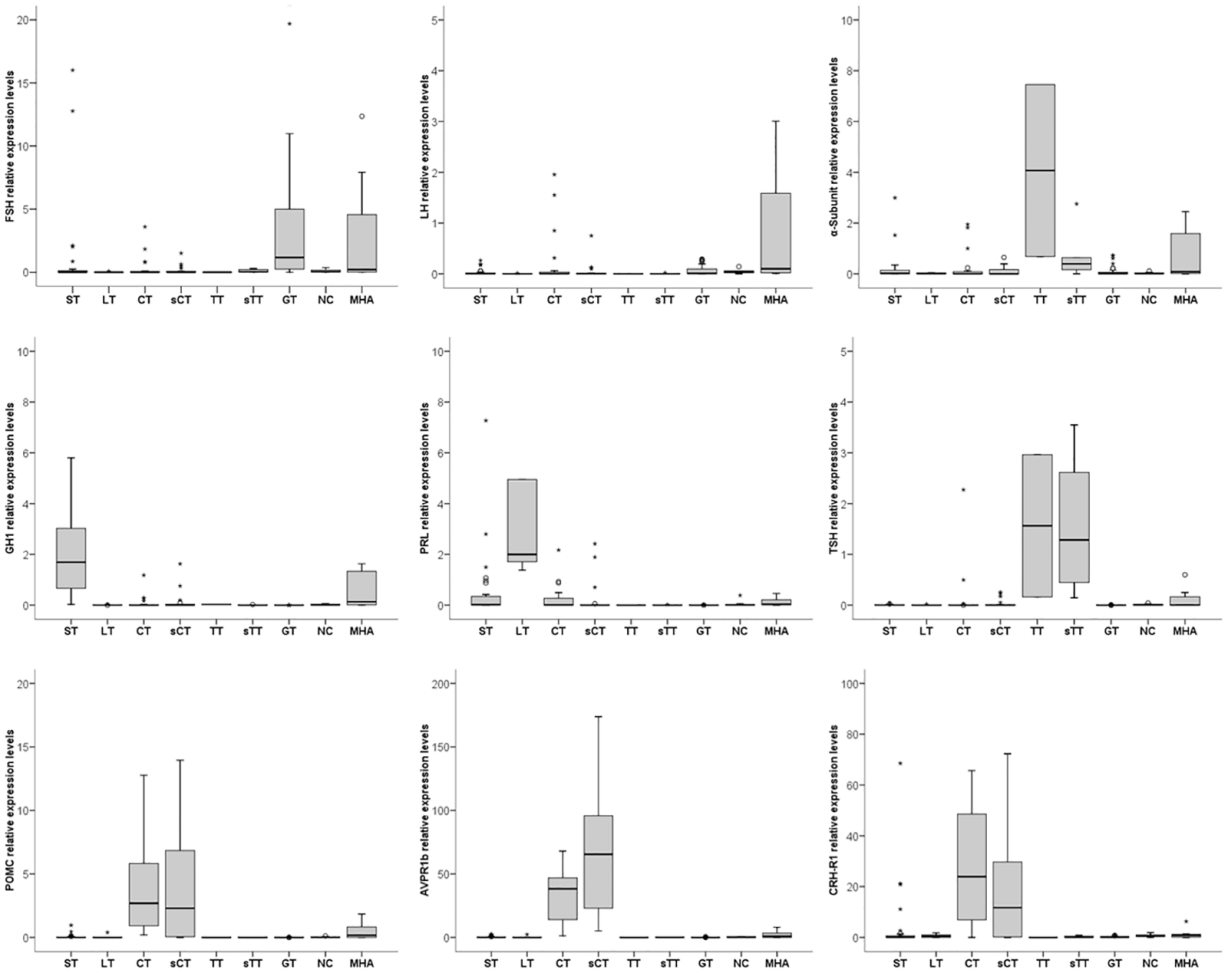
Relative gene expression of hormone and receptor implicated in the molecular classification of pituitary adenomas (n = 142). Figure shows the bar chart of the gene expression levels analyzed for each subtype. Silent lactotroph and silent somatotroph are not represented because there was none in our series.

Although we did not find any molecular sST, sLT or sMHA in our series, we considered these categories because they have been described by others authors [15]. The expression of each hormone and receptor involved in the molecular classification of our series is shown in Fig 1.

### Immunohistochemical studies

The IHC studies were performed in the respective pathology departments of the four participating hospitals. The application of immunohistochemical techniques was performed by equal in the four hospital on complete sections of formalin-fixed tissue and embedded in paraffin in automated form with Autostainer Link48 (Dako-Agilent) equipment, with the Envision high sensitivity display system (Dako). The tissue sections were subjected to dewaxing by incubation in xylol and rehydration using decreasing solutions of ethanol and water. Antigenic recovery was performed with the PT-Link apparatus, treating the sections with target retrieval solution Envision Flex, with high pH (9.0). Primary antibodies were incubated in the Autostainer for 20 to 30 minutes, depending on the setting of the antibodies. Antibodies used and their dilutions in each centre are detailed in “S1 Table”. In all cases immunostaining against the growth hormone, prolactin, adrenocorticotrophin, follicle-stimulating hormone, luteinizing hormone, thyroid-stimulating hormone, and alpha-subunit of pituitary hormones were performed. Antibodies were detected using Envision kit in an automated immunostainer. Depending on the hospital, the IHC results were expressed as either a percentage of positive cells or in semi-quantitative categories (−, +, ++ or +++). To normalize these results, two researchers blinded to clinical information revised the pathology reports and performed a pathological diagnosis, which was used in the study of concordance between molecular and IHC techniques. The criteria used to define the pathological diagnosis was: 1) in case of the results were expressed as a percentage of positive cells, only those with more than 5% were considered positive staining, and 2) in case of semi-quantitative categories only was considered the positive staining regardless of the intensity.

### Molecular analysis

All the molecular studies of the tumours were performed in the Research Unit of the General University Hospital of Alicante.

All specimens were preserved immediately after surgery in RNAlater^®^ solution at 4°C for 24–48 h and then frozen at −80°C until molecular analysis. Molecular methods were adjusted and set up to obtain the highest possible quantity of material from the samples, preserving the quality of the nucleic acids.

Before analysing the study samples, we performed efficiency analyses with two non-valuable samples and two commercial controls of total RNA from brain and total human RNA. The chosen method for extraction was the AllPrep DNA-RNA-Protein (Qiagen). We evaluated the quality and quantity of total RNA in the Nanodrop (Thermo Scientific) and the 2100 Bioanalyzer (Agilent Technologies). The protocol of retrotranscription, selection of primers and quantitative real-time-PCR (qRT-PCR) conditions were adjusted to the material and methods published by Luque et al [12]. Specifically, gene expression levels of all pituitary hormones, type 1 corticotrophin-releasing hormone receptor, arginine vasopressin receptor 1b, and five types of dopamine receptors (*DR1*, *DR4*, *DR5*, *DR2 and its isoform DR2 long*) were measured by qRT-PCR. The results of the dopamine receptors are detailed in “S2 Table”. For normalisation, three housekeeping genes (*GAPDH*, *ACTB and HPRT-1*) were analysed simultaneously and with the same protocol. The reactions were performed in 7500 Fast Real Time PCR System (Applied Biosystems), and results were analysed using SDS software (Applied Biosystems^®^), taking into account the three housekeeping genes. All samples and normal pituitary autopsy samples were analysed in duplicate and following the same protocol. Once normal pituitary autopsy samples were analysed separately and their concordance tested, we mixed them in equal parts into a pool that was analysed as another sample that served as calibrator. A relative quantification was established based on the ΔΔCt method.

### Statistical analysis

Statistical analysis was performed with SPSS 15.0 software (IBM Software). Participant ages and tumour diameters were expressed as mean ± standard deviation (SD). Qualitative variables, including PA subtypes, were expressed as absolute and relative frequencies. Molecular variables showed a non-normal distribution (Kolmogorov-Smirnov test), therefore we chose percentiles as the measure of distributions and reported them as median plus interquartile range (IQR: p25-p75). Cohen's kappa coefficient was calculated to measure inter-classifications agreement (κ = 1 representing complete concordance and κ = 0 null concordance). P values < 0.05 were considered statistically significant.

## Results

### Prevalence of the different subtypes of pituitary adenomas according to identification technique

Clinical and biochemically, 70 patients (49.3%) in our series had a hormonal profile compatible with an endocrine syndrome and were considered as having FPAs, while 72 patients (50.7%) showed normal pituitary function or hypopituitarism and were considered as having NFPAs. The prevalence of the different subtypes according to the IHC or molecular identification is shown in Table 4.

**Table 4:** Prevalence of the different pituitary subtypes according to their inmunohistochemical or molecular identification in the series studied (N = 142).

Pituitary Adenoma Subtype	IHC typification, n (%)	Molecular typification, n (%)
**GT adenomas**	**13 (9.2)**	**46 (32.4)**
FSHomas	6 (46.2)	31 (67.4)
LHomas	4 (30.8)	3 (6.5)
Mixed	3 (23)	12 (26.1)
**CT adenomas**	**17(12)**	**36(25.4)**
*Functioning*	10 (58.8)	19 (52.8)
*Silent*	7 (41.2)	17 (47.2)
**ST adenomas**	**48 (33.8)**	**33(23.2)**
*Functioning*	33 (68.8)	33 (100)
GH	*15 (45*.*5)*	*25 (75*.*7)*
GH-PRL	*16 (48*.*5)*	*3 (9*.*1)*
MH	*2 (6)*	*5 (5*.*2)*
*Silent*	15 (31.2)	-
GH	*7 (46*.*7)*	-
GH-PRL	*8 (53*.*3)*	-
**TT adenomas**	**2 (1.2)**	**7 (4.9)**
*Functioning*	1 (50)	2 (28.6)
*Silent*	1 (50)	5 (71.4)
**LT adenomas**	**11(7.7)**	**5(3.5)**
*Functioning*	8 (72.7)	5 (100)
*Silent*	3 (27.3)	-
**MH adenomas**	**33 (23.2)**	**5 (3.5)**
*Functioning*	14 (42.4)	5 (100)
*Silent*	19 (57.6)	-
**Null Cell adenomas**	**18 (12.7)**	**10 (7)**

PA subtype (GT: gonadotroph, CT: corticotroph, ST: somatotroph, LT: lactotroph, TT: thyrotroph, MH: multihormonal). IHC: inmunohistochemical.

### Reliability of the molecular classification of PA

Once we molecularly identified the different subtypes of PA, we assessed the contribution of the molecular study to the identification of the cellular origin of PA, comparing it with participants’ biochemical and IHC profiles. We contrasted these techniques against the established clinical diagnosis on an individual basis (one per one in all participants).

In the total series (N = 142), the concordance between the three techniques was generally high in all subtypes, with the biochemical study showing the highest kappa values, followed by the molecular analysis **(**Table 5). The concordance between inmunohistochemical and molecular results was different depending on the different centres as shown in “S3 Table”.

**Table 5:** Concordance between clinical diagnosis and independent diagnosis with each technique.

Diagnosis	N	Biochemistry	N	IHC	N	Molecular	N
**NFPA**	74	0.972*	72	0.549	72	0.872	83
**Acromegaly**	36	1.000	36	0.698	48	0.943	33
**Cushing**	21	0.946	23	0.608	10	0.942	19
**Prolactinoma**	9	1.000	9	0.678	11	0.701	5
**Thyrothropinoma**	2	1.000	2	0.664	1	1.000	2

Values show Cohen's kappa coefficient (κ = 1 represents complete concordance and κ = 0 null concordance). All p-values were <0.05. IHC: Immunohistochemistry; NFPA: Nonfunctioning Pituitary Adenomas (including gonadotroph, silent, null cell and multihormonal adenomas).

* Pituitary normofunction.

When we considered only NFPAs (n = 74), the concordance between IHC and molecular profiles was generally low, with the highest level of concordance (κ = 0.519) shown for the sCT subtype and the lowest one (κ = 0.259) for the NC subtype **(**Table 6). It was not possible to calculate the concordance between IHC and molecular profiles on sST, sLT and sMHA subtypes because we did not identify these molecular subtypes in our series.

**Table 6:** Concordance between immunohistochemical and molecular classifications for the 74 clinically NFPA.

NFPA	N IHC	N Molecular	κ	p
**GT**	13	46	0.183	0.014
**NC**	17	6	0.259	0.008
**sCT**	7	17	0.519	0.000
**sTT**	1	5	0.318	0.000
**MHA**	19	0	NP	NP
**sLT**	3	0	NP	NP
**sST**	14	0	NP	NP

Values show Cohen's kappa coefficient (κ = 1 represents complete concordance and κ = 0 the null concordance). All p-values were <0,05. NFPA: Nonfunctioning Pituitary Adenomas. GT: Gonadotroph Adenomas. NC: Null cell adenomas. sCT: Silent Corticotroph Adenomas. MHA: Multihormonal Adenomas. sLT: Silent Lactotroph adenomas. sST: Silent Somatotroph Adenomas. NP: noncalculable parameter.

## Discussion

In this study, we show the molecular typification of a large series of PA. The main strength of the article is the complete clinical, biochemical and immunohistochemical studies of all patients. Moreover, all molecular studies were performed in the same molecular laboratory. Conversely, the main limitation of the study is that the immunohistochemical studies were performed locally in the four University hospitals participants. As immunohistochemistry is a semi-quantitative technique and depends largely of the antibodies chosen, this fact biases significantly the inmunohistochemical results. Indeed, we observed an important variability in the IHC results between the four participating centresmeanwhile in the Lyon’s pathological series, using sensitive IHC techniques, the percentage of NC adenomas were reduced from 10% in 1992 to 1% in 2012 [16]. The variability in the IHC results between the four participating centres is detailed in “S3 Table”.

According to our molecular results, the most prevalent pituitary tumours in our series were GT followed by CT, ST, NC, TT, LT and MHAs. These results were very different from those obtained in the IHC study, where the most prevalent adenomas were the ST, followed by MHA, NC, CT, GT, LT and TT **(**Table 4). The prevalence of LT, both in the IHC and molecular analysis, is underestimated due to the origin of our study sample, as most LTs are not removed surgically. As previously mentioned, the estimated prevalence of pituitary adenoma subtypes has changed frequently, depending on the different series published but also on the technique used in their classification [2].

The classification of PA has also evolved with the advances in pathology. The most important development with clinical implications has been the application of IHC techniques to the characterisation of the tumours, linking the PAs with their protein secretion. IHC techniques have given support to the currently used WHO 2004 classification of PA [8]. However, while there is good concordance between clinical and IHC diagnosis in FPA, the information provided by IHC is more limited in NFPA. Indeed, our results demonstrate that many tumours classified as MHA and NC adenomas immunohistochemically may be reclassified by the molecular study.

NFPAs are frequently macroadenomas and sometimes invasive. The first line of treatment is always surgery, followed when necessary by radiotherapy. In contrast to FPAs, medical treatment of NFPA is mostly ineffective [3]. The prognostic and follow-up of NFPA depends on the results of the mentioned treatments. The overwhelming majority of PAs is benign and slow growing, so if there are no tumour remains after surgery or they have been irradiated, the frequency of clinical follow-up is usually low. However, some NFPA subtypes behave more aggressively than others and should be monitored more closely. This fact has prompted the development of classifications that tailor the prognosis of the PA to the subtype, size, degree of invasiveness and the result of the immunostaining of Ki-67 and p53 [17].

The IHC classification of PA has some limitations. While it represents a spectacular advance in the classification of these tumours, the technique is semi-quantitative and therefore observer-dependent [4,18]. Moreover, immunohistochemistry is an immunometric method that depends on the antibody used. As some PAs secrete different hormone isoforms, they cannot be recognised by specific antibodies. Indeed, Petrus et al. demonstrated that analytical methods using monoclonal antibodies could underestimate FSH levels when the balance of FSH isoforms varied [19]. That could explain why 33 tumours molecularly identified as GT (71.7% of the M-GT), were diagnosed immunohistochemically as NC or other subtypes in our series.

The term ‘null cell adenomas’ has been an important topic of discussion in the literature. Some authors prefer the term ‘non-immunoreactive tumours’ [20], speculating that IHC negativity could correspond to defects in hormone secretion or the production of biologically inactive or insufficient quantities of hormone. In our series, the prevalence of NC adenomas decreased from 12.7% in the IHC classification to 7% in the molecular one. This could be attributed to the higher sensitivity of the molecular technique and the detection of all pituitary hormone isoforms at mRNA level, especially in those tumours without enough protein translation to be detected with the IHC technique.

Similarly, the identification of silent pituitary adenomas was very different between the molecular and the IHC analyses. In our series, a considerable group of NFPA (17/74, 23%) were subclassified as M-sCT, but only 7 (9.5%) of these tumours were confirmed by IHC. The remaining 10 were classified as MHA (n = 4), NC (n = 1), GT (n = 1) and sST adenomas (n = 4). Previous reports in surgical series showed prevalences of sCT between 2.9–5.7% [21,22] similar to our IHC results but clearly inferior to that of the molecular study. The clinical significance of this could be important because it has been suggested that sCT are more aggressive, with higher rates of recurrence, than other PA [23].The silencing of ACTH has been attributed to the production of dysfunctional or high molecular weight ACTH isoforms [24]. Furthermore, Tateno et al. reported a repression of the activity of prohormone convertase (PC) in silent corticotrophs adenomas compared with the functioning CT [25]. PC is the enzyme responsible for the proteolytic processing of POMC in ACTH and other hormones. Therefore, its lack of activity would lead to a defect in the production of ACTH and would explain the negative results of immunostaining and hence the underestimation of the real prevalence of silent corticotropinomas [25,26,27]. Silent corticotropinomas also overexpress the AVPR1b and the CRH-R1 genes, similar to functioning CT[13,14]. The overexpression of these two genes together with the POMC gene in the sCT of our series (Fig 1 and Table 3) corroborated the correct molecular identification of this pituitary tumour subtype.

In the same way, gene expression identified five NFPAs as M-sTT, only one of which was confirmed by IHC. Three cases were identified as MHA (two were positive for TSH, GH and PRL, and one for TSH, GH and FSH). The last one is a discordant case, which we will discuss later.

Eleven sST were identified by the IHC method but not by the molecular analysis. The primer chosen for GH had specificity for all four transcripts of GH, so it is unlikely that the absence of M-sST represented a false negative of the molecular study. Rather, the IHC has a low specificity, attributed to the dilution and the time of incubation with the antibody and to the immunoperoxidase reaction. Authors have highlighted the high number of false positive obtained with this technique [28]. Indeed, one of the IHC-sST was clinically and biochemically diagnosed as Cushing’s syndrome and molecularly identified as M-CT. Nine IHC-sST were identified as M-GT and one as M-NC.

Similarly, four sLT were identified by the IHC method but not by the molecular analysis. Of these, one tumour was molecularly identified as M-CT, and indeed, the patient had Cushing’s syndrome. This case represents, as above, a false positive from the IHC analysis. The other three IHC-sLT were molecularly identified as M-GT.

The molecular identification of PAs proposed in the present article has shown to be highly robust to the cellular origin of these tumours. The molecular characterisation is quantitative and easily standardisable technique, so it has lower variability and higher specificity and sensitivity than the IHC technique. Indeed, the molecular classification of PA was always more consistent with the clinical manifestations than IHC **(**Table 4). Moreover, the molecular analysis allowed the re-classification of an important number of silent and IHC-NC tumours.

The clinical significance of molecular PA identification has not been yet established. As previously said, it is possible that M-sCT could behave with the same aggressiveness as IHC-sCT. Moreover, molecular silent tumours expressing PRL, TSH or GH genes could reduce their size if be treated with cabergoline or somatostatin analogues. Besides, the number of anti-angiogenic drugs is increasing daily, and we have recently reported that the angiogenic pathway largely depends on the cellular origin of the PA [29]. In this scenario, the complete identification of the cellular origin of PA could be essential in achieving optimal results.

The molecular classification of PA tumours also has some limitations. The mechanisms behind the silencing of some types of tumours and its clinical relevance are still unclear. Moreover, the pre-operative treatment of the tumour can affect gene expression. While in our series no tumour was irradiated before the molecular study, some patients had received prior medical treatment **(**Table 1). We observed that all LT treated previously with cabergoline showed lower than expected PRL gene expression levels (0.01 FC to 2.00 FC). In fact, in the present series there were some discrepant cases between the molecular, clinical and IHC diagnosis:

1. One patient with NFPA with positive immunostaining for ACTH and PRL was molecularly identified as M-TT. The patient suffered from primary hypothyroidism in substitution treatment with levothyroxine. Although previous studies have reported that patients with non-controlled primary hypothyroidism could develop TT [30], hormonal replacement usually reverses this condition. Therefore, we consider that this tumour is a sTT and that the IHC results were false positives.
2. Three patients with gene expression profile of M-GT and clinically diagnosed as NFPA had positive immunostaining for PRL. Biochemically, only one of these patients had mild hyperprolactinaemia (42.5 ng/mL), which was attributed to a lesion of the dopaminergic pathways. Therefore, we consider again that all three of these cases are probably false positives of the IHC technique.
3. One patient with clinical, biochemical and IHC diagnosis of LT did not express the PRL gene. However, the expression of dopamine receptors DR2 and DR4 in this tumour resembled that of the LT adenomas. Moreover, the tumour overexpressed the receptors DR1 and DR5 (50 and 21 FC respectively). This patient had been previously treated with high doses of cabergoline (3.5 mg/week) for four years. The most plausible explanation was that the long-term treatment with cabergoline downregulated the PRL gene and increased the DR1 and DR5 gene expression, as reported elsewhere [31,32].
4. One patient with clinical and biochemical diagnosis of LT expressed neither PRL protein nor the PRL gene and was erroneously identified via IHC and molecularly as having NC adenoma. Once again, the patient had been treated with cabergoline for a long time (more than four years). It is possible that different degrees of downregulation have sequential effects on the gene and the protein expression. In fact, other authors had shown that treatment with dopamine agonists can decrease PRL and its receptor mRNA levels [33]. Because of the complexity of the regulatory role of dopamine agonists on PRL cell function, many more studies are needed to elucidate the mechanisms by which they affect to PRL mRNA or protein levels.
5. One patient molecularly classified as M-ST had a clinical LT and showed positive immunostaining for all pituitary hormones. He was a 14-year-old patient with macroprolactinoma, in treatment with cabergoline for four years. Despite the normalisation of the PRL levels, the boy had a growth and puberty delay. The tumour expressed the GH gene at high levels (more than 3 FC) without gene expression of PRL. The most plausible explanation is that the sample analysed was normal pituitary, with a high growth hormone expression corresponding to adolescence and a lack of PRL gene expression due to the cabergoline treatment. This case highlights the need for a pathologist to select the tissue for analysis before performing any molecular study.

## Conclusions

In this large series, molecular profiling showed higher accuracy than the immunohistochemical study in the identification of pituitary adenomas subtypes. This finding entails changes in the known prevalence of these tumours with possible clinical repercussions. Indeed, the prevalence of silent CT, a tumour PA subtype that often behaves aggressively, increases significantly with the molecular compared to IHC techniques. Moreover, gene expression allows reclassification of a large part of the IHC null cell adenomas. Therefore, the molecular profiling complements the pathological information of the IHC in the study of PA, although previous medical treatments may act as confounders by modifying the gene expression. Even though the not centralized performance of IHC analysis in this study could bias the IHC results, this fact increases the value of the molecular typification of PA as complement of the IHQ information in NFPA. However, it is worth highlighting the importance that an expert pathologist carefully identifies the used tissue for molecular analysis.

## Supporting information

S1 Table Antibodies used in immunohistochemical studies performed in pathology departments of the four participating hospitals. (DOC)

S2 Table Relative expression [median (p25-p75)] of dopamine receptors (DR) *DR1*, *DR4*, *DR5*, *DR2* and its isoform *DR2 long*, according to molecular subtypes of pituitary adenomas. This is the S2 Table legend: PA: pituitary adenomas, DR: dopamine receptors, NC: null cell adenomas, GT: gonadotroph adenomas, CT: corticotroph adenomas, sCT: silent corticotroph adenomas, ST: somatotroph adenomas, LT: lactotroph adenomas, TT: thyrotroph adenomas, sTT: silent thyrotroph adenomas.
(DOC)

S3 Table Concordance between clinical and immunohistochemical / molecular diagnosis in each hospital participating in the study. This is the S3 Table legend: Values show Cohen's kappa coefficient (κ = 1 represents complete concordance and κ = 0 the null concordance). All p-values were 0,000. IHC: Immunohistochemistry; H: Hospital; NFPA: Nonfunctioning Pituitary Adenomas; NC: non-calculable.
(DOCX)
