# Supplementary material for: Contribution of molecular analysis to the typification of the non-functioning pituitary adenomas
Source: PLoS One. 2017 Jul 10;12(7):e0180039. doi: 10.1371/journal.pone.0180039 (PMC5503173; doi:10.1371/journal.pone.0180039)
Supplement: S1 Table — (DOC) [file pone.0180039.s001.doc]

S1 Table. Antibodies used in immunohistochemical studies performed in pathology departments of the four participating hospitals.

| HORMONE | ANTIBODY USED | DILUTION |
| --- | --- | --- |
| **Follicle-stimulating Hormone** |  |  |
| Hospital 1 | Monoclonal Mouse Anti-Human FSH  Clone C10 – M3504 DAKO | 1:200 |
| Hospital 2 | FSH Menarini policlonal | 1:50 |
| Hospital 3 | Monoclonal Mouse Anti-Human FSH  Clone C10 – M3504 DAKO | 1:50 |
| Hospital 4 | Monoclonal Mouse Anti-Human FSH  Clone C10 – M3504 DAKO | 1:50 |
| **Luteinising Hormone** |  |  |
| Hospital 1 | Monoclonal Mouse Anti-Human LH  Clone C93 – M3502 DAKO | 1:300 |
| Hospital 2 | LH Menarini policlonal | 1:50 |
| Hospital 3 | Monoclonal Mouse Anti-Human LH  Clone C93 – M3502 DAKO | 1:50 |
| Hospital 4 | Monoclonal Mouse Anti-Human LH  Clone C93 – M3502 DAKO | 1:50 |
| **Alpha-subunit** |  |  |
| Hospital 1 | 4E12. Novocastra | 1:100 |
| Hospitals 2-4 | Not done |  |
| **Prolactin** |  |  |
| Hospital 1 | Polyclonal Rabbit Anti-Human Prolactin  DAKO A0569 | 1:200 |
| Hospital 2 | Polyclonal Rabbit Anti-Human Prolactin  DAKO A0569 | 1:50 |
| Hospital 3 | Polyclonal Rabbit Anti-Human Prolactin  DAKO A0569 | 1:200 |
| Hospital 4 | Biomeda | 1:200 |
| **Adrenocorticotropic Hormone** |  |  |
| Hospital 1 | Monoclonal Mouse Anti- ACTH  Clone 02A3 – M3501 DAKO | 1:500 |
| Hospital 2 | Monoclonal Mouse Anti- ACTH  Clone 02A3 – M3501 DAKO | 1:50 |
| Hospital 3 | Monoclonal Mouse Anti- ACTH  Clone 02A3 – M3501 DAKO | 1:100 |
| Hospital 4 | Monoclonal Mouse Anti- ACTH  Clone 02A3 – M3501 DAKO | 1:100 |
| **Growth Hormone** |  |  |
| Hospital 1 | Polyclonal, Novocastra | 1:100 |
| Hospital 2 | HGH Dako polyclonal | 1:50 |
| Hospital 3 | HGH Dako polyclonal | 1:50 |
| Hospital 4 | HGH Dako polyclonal | 1:300 |
| **Thyroid- stimulating Hormone** |  |  |
| Hospital 1 | Monoclonal Mouse Anti-Human TSH  Clone 0042 – M3503 DAKO | 1:300 |
| Hospital 2 | Monoclonal Mouse Anti-Human TSH  Clone 0042 – M3503 DAKO | 1:50 |
| Hospital 3 | Monoclonal Mouse Anti-Human TSH  Clone 0042 – M3503 DAKO | 1:50 |
| Hospital 4 | Biomeda | 1:100 |
