# Supplementary material for: Contribution of molecular analysis to the typification of the non-functioning pituitary adenomas
Source: PLoS One. 2017 Jul 10;12(7):e0180039. doi: 10.1371/journal.pone.0180039 (PMC5503173; doi:10.1371/journal.pone.0180039)
Supplement: S2 Table — This is the S2 Table legend: PA: pituitary adenomas, DR: dopamine receptors, NC: null cell adenomas, GT: gonadotroph adenomas, CT: corticotroph adenomas, sCT: silent corticotroph adenomas, ST: somatotroph adenomas, LT: lactotroph adenomas, TT: thyrotroph adenomas, sTT: silent thyrotroph adenomas. (DOC) [file pone.0180039.s002.doc]

**S2 Table**. **Relative expression [median (p25-p75)] of dopamine receptors *DR1, DR4, DR5, DR2* and its isoform *DR2 long*, according to molecular subtypes of pituitary adenomas.**

| **Molecular PA Subtype** | ***DR1*** | ***DR4*** | ***DR5*** |
| --- | --- | --- | --- |
| **NC** | 1.627 (0.092-11.582) | 1.144 (0.143-2.348) | 0.021 (0.011-0.08) |
| **GT** | 0.446 (0.111-2.666) | 0.675 (0.396-1.106) | 0.046 (0.000-0.223) |
| **CT** | 0.000 (0.000-0.3275) | 1.105 (0.539-18.405) | 0.014 (0.000-0.030) |
| **sCT** | 0.071 (0.000-0.228) | 1.517 (0.561-1.95) | 0.001 (0.000-0.027) |
| **ST** | 0.224 (0.000-1.538) | 0.897 (0.553-1.386) | 0.052 (0.041-0.080) |
| **LT** | 0.000 (0.000-10.735) | 0.647 (0.582-1.389) | 0.014 (0.000-0.062) |
| **TT** | 6.616 (0.229-13.003) | 2.188 (1.083-3.293) | 0.053 (0.022-0.083) |
| **sTT** | 3.49 (0.670-97.172) | 1.097 (0.577-1.802) | 0.214 (0.024-2.174) |
| **Mh** | 0.453 (0.319-1.506) | 11.905 (0.595-19.995) | 0.088 (0.022-0.511) |
|  | ***DR2*** | ***DR2 long*** |  |
| **NC** | 0.489 (0.002-0.767) | 0.321 (0.002-0.433) |  |
| **GT** | 0.638 (0.450-0.924) | 0.511 (0.324-0.921) |  |
| **CT** | 0.153 (0.037-0.415) | 0.155 (0.026-0.394) |  |
| **sCT** | 0.128 (0.008-0.610) | 0.121 (0.008-0.591) |  |
| **ST** | 0.309 (0.157-0.570) | 0.278 (0.146-0.521) |  |
| **LT** | 2.299 (0.892-4.318) | 1.973 (0.954-31.815) |  |
| **TT** | 0.176 (0.162-0.190) | 0.130 (0.087-0.172) |  |
| **sTT** | 0.513 (0.071-1.000) | 0.410 (0.071-0.811) |  |
| **Mh** | 0.796 (0.480-1.133) | 0.791 (0.438-1.085) |  |

PA: pituitary adenomas, DR: dopamine receptors, NC: null cell adenomas, GT: gonadotroph adenomas, CT: corticotroph adenomas, sCT: silent corticotroph adenomas, ST: somatotroph adenomas, LT: lactotroph adenomas, TT: thyrotroph adenomas, sTT: silent thyrotroph adenomas, Mh: multihormonal adenomas.
