# Supplementary material for: Contribution of molecular analysis to the typification of the non-functioning pituitary adenomas
Source: PLoS One. 2017 Jul 10;12(7):e0180039. doi: 10.1371/journal.pone.0180039 (PMC5503173; doi:10.1371/journal.pone.0180039)
Supplement: S3 Table — This is the S3 Table legend: Values show Cohen's kappa coefficient (κ = 1 represents complete concordance and κ = 0 the null concordance). All p-values were 0,000. IHC: Immunohistochemistry; H: Hospital; NFPA: Nonfunctioning Pituitary Adenomas; NC: non-calculable. (DOCX) [file pone.0180039.s003.docx]

**Table S3. Concordance between clinical and immunohistochemical / molecular diagnosis in each hospital participating in the study.**

| **Diagnosis** | **NFPA** | **Acromegaly** | **Cushing** | **Prolactinoma** | **Thyrothropinoma** |
| --- | --- | --- | --- | --- | --- |
| N | 86 | 42 | 22 | 8 | 2 |
| **IHC** | **0,413** | **0,678** | **0,563** | **0,527** | **0,664** |
| H1 | 0,930 | 0,910 | NC | 1,0 | 1,0 |
| H2 | 0,310 | 0,660 | 0,507 | 0,417 | NC |
| H3 | 0,060 | 0,600 | 1,0 | NC | NC |
| H4 | 0,125 | 0,458 | 0,660 | -0,091 | NC |
| **Molecular** | **0,886** | **0,932** | **0,916** | **0,552** | **1,000** |
| H1 | 0,917 | 0,917 | NC | 0,469 | 1,0 |
| H2 | 0,916 | 0,922 | 0,929 | 0,556 | 1,000 |
| H3 | 0,700 | 1,0 | 1,0 | NC | NC |
| H4 | 0,792 | 1,0 | 1,0 | 0,643 | NC |
| Values show Cohen's kappa coefficient (κ=1 represents complete concordance and κ=0 the null concordance). All p-values were 0,000. IHC: Immunohistochemistry; H: Hospital; NFPA: Non-functioning Pituitary Adenomas; NC: non-calculable. | | | | | |
